# Acute Kidney Injury: Magnitude and Predictors of Maternal Outcomes among Pre-Eclamptic and Eclamptic Women in Mwanza, Tanzania

**DOI:** 10.24248/eahrj.v8i3.807

**Published:** 2025-01-30

**Authors:** Mathias Njau, Fridolin Mujuni, Dismas Matovelo, Edgar Ndaboine, Richard Kiritta, Ladius Rudovick

**Affiliations:** a Department of Obstetrics and Gynaecology, Catholic University of Health and Allied Sciences, Mwanza, Tanzania; b Department of Internal Medicine-Nephrology Unit, Catholic University of Health and Allied Sciences, Mwanza, Tanzania

## Abstract

**Background:**

Acute kidney injury (AKI) that occurs during pregnancy or in the post-partum period is a serious obstetric complication with a significant risk of feto-maternal morbidity and mortality. Although globally there has been an overall decrease in the burden of pregnancy-related (PRAKI) AKI, recent studies show the increasing occurrence of this disease in Low-middle income countries (LMICs).

This study was conducted to determine the magnitude and predictors of maternal outcomes of pre-eclamptic and eclamptic-related acute kidney injury at Bugando Medical Centre (BMC) and Sengerema Designated District Hospital (SDDH) Mwanza, Tanzania.

**Methods:**

A cohort study was conducted involving patients diagnosed with Preeclampsia-Eclampsia (PE-E) who were admitted at BMC and SDDH from November 2021 to June 2022. Data were collected through convenience sampling using a structured questionnaire. Statistical data analysis was performed using STATA version 15. A chi-square test was performed to test for significant associations between the predictor and outcome variables. A significant association was defined as a p-value of less than .05. Variables showing significant associations in the chi-square analysis were further analysed using multivariate logistic regression to evaluate the strength of the associations.

**Results:**

The study enrolled a total of 258 women with PE-E. The magnitude of AKI was found to be 141(54.7%,) out of 258 participants, of which Kidney Disease Improving Outcomes (KDIGO) stage 1 accounted for 103 (73%) stage 2, 20 (14.2%) and stage 3, were 18 (12.8%). Of these, 141 participants with AKI, 101 (71.6%) resolved within 7 days of delivery, 12 (8.5%) persisted beyond 7 days after delivery, and 28 (19.8%) worsened. Serum creatinine stages 2 and 3 at admission, HIV positive status, and informal education status were highly associated with persistent/worsening AKI stage.

**Conclusion:**

The magnitude of acute kidney injury was found to be high (54.7%) among pre-eclamptic and eclamptic patients in BMC and SDDH. AKI stages 2 and 3, HIV positive status and informal education level were associated with poor maternal outcomes mainly persistent AKI and worsening AKI.

## BACKGROUND

Acute kidney injury is defined as the deterioration of kidney function resulting in the accumulation of serum creatinine and urea. As per KDIGO staging criteria, it is an increase in serum creatinine by >0.3mg/dl within 48 hours or an increase in serum creatinine to ≥ 1.5 times baseline which is known or presumed to have occurred within the prior7 days, or urine volume < 0.5mls/kg/hour for 6 hours. Pregnancy-Related Acute Kidney Injury (PRAKI) was defined as Acute Kidney Injury (AKI) diagnosed anytime during pregnancy or during the postpartum period.

Globally, preeclampsia-eclampsia (PE-E) accounts for 2 to 8% of all pregnancies and 15% to 20% of PRAKI cases and is the leading cause of AKI worldwide.^1,2^ It is further a common cause of AKI in low- and middle-income countries (LMICs).^3^ It primarily presents as a rapid decline in kidney filtration function that results in the retention of urea and other nitrogenous waste products, and dysregulation of extracellular volume and electrolytes.^4,5^ In pregnancy, AKI usually occurs in women with previously healthy kidneys, but it can complicate the course of patients with pre-existing renal disease.^5^

Worldwide, the burden of PRAKI has decreased markedly during the second half of the 20^th^ century, due to improvement in obstetric and prenatal care as well as a decline in the rate of unsafe abortion in High-Income Countries (HICs).^6^ Over decades, the worldwide incidence of PRAKI has decreased from 20% to 40% in 1960 to less than 10% in recent years, this is probably due to improvements in antenatal care, the legalization of abortion, and improved obstetrics care.^7^ In recent years, the burden of AKI in pregnancy has decreased in HICs to only 1% to 2.8%.^8^ However, pregnancy-related AKI is still frequent in Low Middle-Income Countries (LMICs); where the burden is around 15 to 20%.^8^ The high burden in LMICs is mainly due to limited access to emergency obstetric healthcare.^9^ Several studies reported maternal mortality from PRAKI to range between 20% to 67% which is higher than earlier reports of 9% to 55%.^10^

The timing of AKI during pregnancy may serve as an important clue as to the underlying aetiology. The vast majority of AKI occurs in the second and third trimesters mainly due to hypertensive complications such as PE-E.^11^ Although the aetiology of PRAKI varies between countries, PE-E accounts for 5 to 20% of cases^12^ with one study in LMICs reporting 36% of PRAKI to be due to hypertensive disorders of pregnancy.^13,14^ According to the study done by Ndaboine et al 2012 at Bugando Medical Centre (BMC), Tanzania reported that the incidence of eclampsia was 1.37% and among them, 10.5% had AKI.^15^ The study done by Israel et al 2017 at Dodoma Referral Regional Hospital, reported that the prevalence of AKI in PE-E patients was 5.1% and 8.3% respectively.^16^

Even though the burden of PRAKI has been declining in HICs, it remains a serious challenge in LMICs due to its association with significant adverse maternal and foetal outcomes.^17–19^ Concerning the outcome of this condition, complete renal recovery is commonly achieved if these patients receive appropriate and timely management. Fewer patients may become entirely dependent upon dialysis due to inadequate initial resuscitation, longer intervals to reach an appropriate clinical setting, and consequently, significantly late initiation of dialysis.^19^

However, the predictors of AKI among pregnant and postpartum women with PE-E in Tanzania as well as its impact on maternal outcomes remain inadequately studied. Additionally, data on AKI associated with PE-E is scarce, and its prevalence and outcomes in affected mothers are largely unknown.. Thus, this study was designed to determine the magnitude and predictors of maternal outcomes of PE-E-related AKI at BMC in Tanzania and Sengerema District Designated Hospital (SDDH).

## METHOD AND MATERIALS

### Study Design, Population Setting, and Duration

**Study Design and Setting:** This was a cohort study involving 258 women with PE-E at the maternity and gynaecology ward at BMC and SDDH for 8 months (November 2021 to June 2022). Bugando Medical Centre (BMC) is a consultant, tertiary care and teaching hospital for the Catholic University of Health and Allied Sciences-Bugando (CUHAS-Bugando) located in Mwanza City, north-western Tanzania. Has a bed capacity of 960 beds. Department of Obstetrics and gynaecology alone has a bed capacity of 128. BMC serves as a referral centre for tertiary specialist care for a catchment population of approximately 17 million people from neighbouring regions.

Sengerema district-designated Hospital (SDDH) is a large mission hospital in Sengerema, Mwanza province-Tanzania that was founded in 1959 by the brothers of St. Johannes de Deo and the nuns of St. Charles Borromeo. The hospital has around 300 beds, distributed over 9 wards. The Department of Obstetrics and gynaecology wards alone has a bed capacity of 61 beds (maternity ward 31 beds, gynaecology ward 16 beds and labour ward 14 beds).

### Study Population

Consent was obtained from patients aged 18 years and above for their enrollment in the study. For patients under 18 years or those who were severely ill, assent was sought from their parents or guardians.

The study population comprised patients with PE/E, with or without acute kidney injury, who were admitted to BMC and SDDH during the study period. Consent was obtained from patients aged 18 years and above for their enrolment in the study. For patients under 18 years or those who were severely ill, assent was sought from their parents or guardian.

### Sampling Procedure

Participants were selected through convenience sampling, and those meeting the inclusion criteria were enrolled until the target sample size was achieved.

### Inclusion Criteria

All patients with a gestational age of more than 20 weeks, admitted with a clinical diagnosis of preeclampsia or eclampsia, and who had blood samples taken for biochemical tests during admission were included.

### Exclusion Criteria

Patients with known pre-existing renal disease or renal insufficiency before pregnancy were excluded from the study. Those who refused to consent were also not included.

### Sample Size

The minimum sample size was calculated by using the DANIEL 1999 formula.


$$\text{n}={\text{Z}}_{\text{x}}^{2}\text{ P}(1-\text{P})/{\text{d}}^{2}$$

Where by n= sample size, Z = value from standard normal distribution corresponding to desired confidence interval (Z= 1.96 for 95% CI), *P*= was considered as 10.5% as prevalence of AKI from the study done by Ndaboine et al^15^ among eclamptic patients at Bugando Medical Centre, d= desired precision 0.05

### Independent Variables

Maternal age, area of residency, history of hypertensive disorder before or in previous pregnancies, body mass index, HIV status, education status, parity status, and renal status at admission, protein in urine at admission, gestation age at admission, admission systolic blood pressure and admission diastolic blood pressure.

### Dependent Variables

Resolving AKI, persisting AKI, and worsening AKI.

### Data Collection and Laboratory Analysis

Pre-tested structured questionnaires were used to collect participants ‘information. For those who met the criteria, but were unable to respond, their next of kin provided the required information. Blood samples for renal biochemistry (serum creatinine and urea) were collected by the investigator or a trained assistant. Serum creatinine was measured using the Jaffe rate method (kinetic alkaline picrate). A precise volume of the sample was introduced into a reaction cup containing an alkaline picrate solution, and absorbance readings were taken at 552 nm between 19 and 25 seconds after sample injection. Creatinine in the sample reacted with the reagent to produce a red-coloured complex, with the absorbance rate directly corresponding to the creatinine concentration. The analysis was performed using the COBAS Integra 400 Plus Analyser, (Roche Diagnostics Ltd, Rotkreuz) in the ISO Certified Bugando Medical Centre Laboratory (ISO 15189:2012). After sample analysis, participants continued to receive treatment.

Participants were monitored for changes in serum creatinine levels on day 3 (72 hours) and day 7, or earlier if discharged. Consent was obtained from patients before enrolment, and assent was sought from their guardians when applicable. Confidentiality was maintained throughout the study.

### Diagnosis of AKI

As per KDIGO staging criteria, AKI is defined as an increase in serum creatinine by >0.3mg/dl within 48 hours or an increase in serum creatinine to ≥ 1.5 times the baseline value (known or presumed to have occurred within the previous 7 days), or a urine volume < 0.5mls/kg/hour for 6 hours.

### Data Analysis

Statistical data analysis was performed using STATA version 15 (College Station, Texas, USA). Categorical variables were summarised using proportions and frequency tables. Continuous variables were reported as mean ± standard deviation (SD) or interquartile range IQR. A chi-square test was performed to test for significant associations between the predictor and outcome variables. A significant association was defined as a *p*-value of less than 0.05. Further analysis by logistic regression was done for those variables which were significant on chi-square.

### Ethical Considerations and Consent

The study was cleared by the CUHAS/BMC Research Ethics and Review Committee with certificate number CREC/506/2021. Permission to conduct this study was requested from the hospital authority (BMC and SDDH) with reference later AB.286/317/01. Enrolled patients signed a written informed consent for the study. Patients’ identity and information obtained from this study was not disclosed and were assured for confidentiality. The study did not interfere with the decision of the attending doctors, that patient who needed dialysis, and financially were not capable assistance from the social welfare was sought and treatment continued. Those patients who refused to consent and those who withdraw from the study continued to access medical services without any compromise. All methods were performed according to Declaration of Helsinki.

## RESULTS

### Social Demographic Characteristics of Patients

The screening was done from obstetrics and gynaecology admissions during the study period and 258 participants were enrolled out of 3742 admissions.

Most of the study participants, 128 (49.6%), were between the ages of 26 and 35. The youngest was 16 and the oldest was 43, with a mean age of 29.53 SD 6.62. Most of the participants were referrals from other facilities 138(53.5%). The majority of the participants were living in urban areas 210(81.4%) and most of them had primary education 140 (54.3%). Two hundred and thirty-five (91.1%) were married and 231(89.5%) were unemployed. (Table 1)

**Table 1:** Demographic Characteristics of Patients

Variable	Frequency (n)	Percentage (%)
Age category		
16–25 years	73	28.3
26–35 years	128	49.6
≥36 years	57	22.1
Referral status		
Self–referral	120	46.5
Referral from other	138	53.5
Residence		
Urban	210	81.4
Rural	48	18.6
Education		
Informal education	15	5.8
Primary education	140	54.3
Secondary education	68	26.4
College Education	35	13.6
Occupation		
Employed	27	10.5
Unemployed	231	89.5
Marital status		
Single	23	8.9
Married	235	91.1

### Maternal Clinical Characteristics

The majority of participants had a previous history of hypertension 147 (57.0%), and 159 (61.6%) were overweight. The majority of participants 160 (62.0%) had late-onset pre-eclampsia. Urine for protein test 2 plus and above in 246 (95.4%), and severe systolic BP were 122 (47.3%) while severe diastolic BP was 161 (62.4%). (Table 2)

**Table 2:** Maternal Clinical Characteristics

Variable	Frequency (N)	Percentage (%)
History of hypertension in the-previous pregnancy		
Yes	147	57.0
No	111	43.0
Body mass index.		
Normal weight	81	31.4
Overweight/obese	177	68.6
HIV status		
Reactive	12	4.7
Non-reactive	246	95.4
Gravidity category		
Primegravida	71	27.5
Multigravida	187	72.5
Parity category		
Low parity (1–3)	202	78.3
High parity (≥4)	56	21.7
Preeclampsia onset		
Early onset	98	38.0
Late-onset	160	62.0
Hypertension severity (SBP)		
Mild (SBP 140–159mmhg)	136	52.7
Severe (SBP ≥160mmhg)	122	47.3
Hypertension severity (DBP)		
mild (90–109mmhg)	161	62.4
severe (≥110mmhg)	97	37.6
Urine protein		
+	12	4.7
≥++	246	95.4
Time from diagnosis of preeclampsia-to delivery (intervention)		
Early (within 48hours)	198	76.7
Late (after 48hours)	60	23.3

### Serum Creatinine Status at Admission

**Figure 1:**
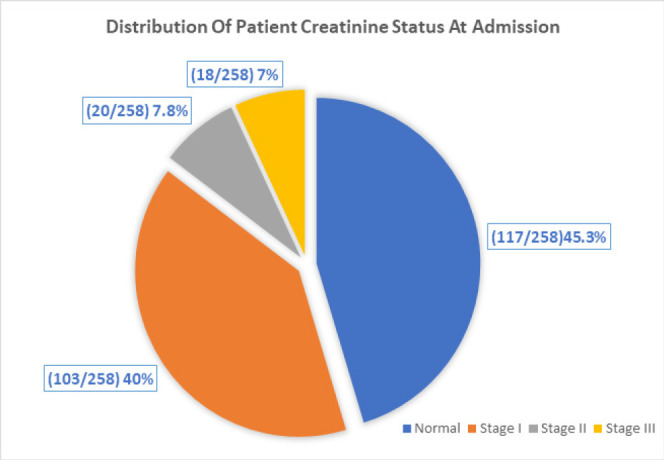
Distribution of AKI Stage of Presentation Among Preeclamptic/Eclamptic Participants with Elevated Serum Creatinine. N-141

Among the 258 participants who were diagnosed with pre-eclampsia/eclampsia, 117 (45.3%) had normal kidney function while 141 (54.7%) had AKI. And among those with AKI, 103 (73%) had stage I AKI, 20 (14.2%) were at stage II and 18 (12.8%) were at stage III AKI. (Figure 1)

### Time from the Diagnosis of Preeclampsia/Eclampsia to Intervention (i.e., Referral, Delivery)

The majority of the participants 198 (76.7%) had early intervention (i.e., they were intervened within the same week at diagnosis of preeclampsia) while 60 (23.3%) had late intervention (i.e., more than one week from Gestation Age (GA) at which preeclampsia was diagnosed).

### Distribution of Outcomes of Preeclampsia/Eclampsia-related AKI

**Figure 2:**
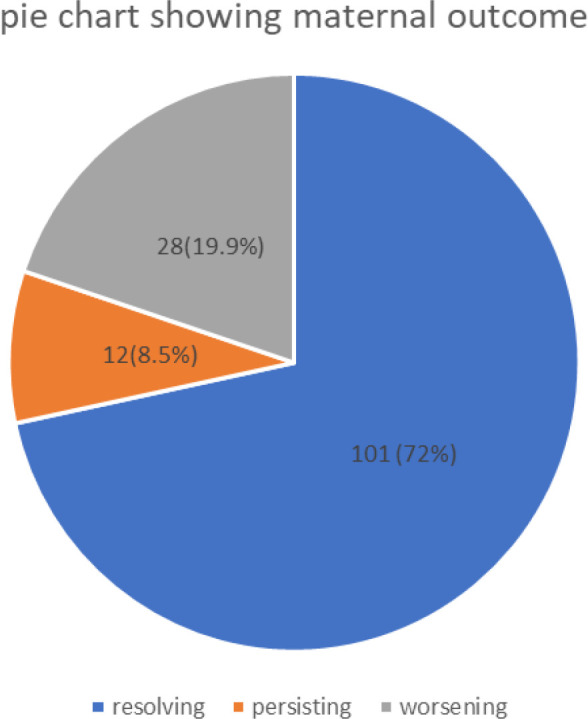
Distribution of Outcomes of Preeclampsia/Eclampsia-Related AKI

Of the 141 participants who had preeclampsia/eclampsia-related AKI, 101 (72%) had AKI which resolved to normal within 7 days post-delivery, 12(8.5%) persisted with elevated serum creatinine, while 28 (19.9%) worsened. (Figure 2)

### Status of the Participants at the end of follow-up

The majority of the participants 219(84.9%) were managed conservatively and discharged home, (this group included those with preeclampsia with no AKI and those with stage 1 AKI with no severe features) while 29(11.2%) needed dialysis and 10(3.9%) died after the seventh day of follow up. The association between maternal characteristics and outcome of preeclampsia/eclampsia-related acute kidney injury

Upon analysing to see the association through chi-square, age, residence, BMI, SBP, and DBP, urine for protein, Parity, and Gestation age at onset all showed no association. History of previous hypertension (*p*-value .025), HIV positive (*p*-value .003), high serum Creatinine levels (*p*-value <.001), and informal Education level (*p*-value .008) showed an association. These were further run through multivariate analysis and only 3 came out with an association with maternal outcome (informal education level, high serum creatinine level, and HIV positive). (Table 3)

**Table 3:** Association of Maternal Clinical Characteristics and Outcomes

Variable	Outcomes		Total	Chi2	*P-value*	Multivariate	
	Resolving n (%)	Not resolving n (%)				OR [95% CI]	*P-value*
Age category							
16–25	32 (32)	11 (26.8)	43 (30.5)	0.4460	0.800		
26–35	48 (48)	22 (53.7)	70 (49.7)				
≥ 36	20 (20)	8 (19.5)	28 (19.9)				
Area of residency							
Urban	80 (80)	29 (70.7)	109 (77.3)	1.4237	0.233		
Rural	20 (20)	12 (29.3)	32 (22.7)				
History of hypertension in previous							
Yes	73 (73)	37 (90.2)	110 (78.0)	5.0410	0.025	2.47 (0.67–9.06)	0.172
No	27 (27)	4 (9.8)	31 (22)		1		
Body mass index (BMI) category							
Normal weight	19 (19)	4 (9.8)	23 (16.31)	1.8201	0.177		
Abnormal weight	81 (81)	37 (90.2)	118 (83.7)				
HIV							
Non-reactive	97 (97)	34 (82.9)	131 (92.9)	8.7401	0.003	1	
Reactive	3 (3.0)	7 (17.1)	10 (7.1)			6.49 (1.23–34.24)	0.034
Serum creatinine category at admission							
Stage I	86 (86.0)	16 (39.02)	102 (72.34)	32.8411	<0.001	1	
Stage II	10 (10)	9 (21.95)	19 (13.5)			2.90 (.86–9.82)	0.085
Stage III	4 (4)	14 (34.15)	18 (12.8)			21.56 (5.36–86.72)	<0.001
Systolic Bp category							
≥140–≤159mmhg	19 (19)	4 (9.8)	23 (16.3)	1.8201	0.177		
≥160mmhg	81 (81)	37 (90.24)	118 (83.7)				
Diastolic Bp							
90–109	61 (61)	24 (58.5)	85 (60.3)	0.0737	0.786		
≥110	39 (39)	17 (41.5)	56 (39.7)				
Education							
Informal	4 (4)	7 (17.1)	11 (7.8)	11.7966	0.008	36.09 (2.27–573.48)	0.011
Primary	53 (53)	25 (25.98)	78 (55.32)			10.20 (.94–109.95)	0.056
Secondary	27 (27)	8 (19.51)	35 (24.82)			9.93 (.82–119.54)	0.070
College	16 (16.00)	1 (2.44)	17 (12.1)			1	
Urine for Protein							
+	1 (1.00)	2 (4.9)	3 (2.13)	2.1000	0.147		
≥ ++,	54 (76.1)	17 (23.9)	71 (100)				
Parity category							
Low parity	81 (81)	28 (68)	109 (77.3)	2.6763	0.102		
High parity	19 (19)	13 (31.7)	32 (22.7)				
Gestation age at diagnosis							
Early onset	99 (99)	41 (100)	140 (99.3)	0.4129	0.520		
Late-onset	1 (1–0)	0 (0)	1 (0)				
Time from diagnosis of preeclampsia to delivery							
Early within 48hours	80 (80)	28 (68.3)	108 (76.6)	2.2232	0.136		
Late after 48hours	20 (20)	13 (31.7)	33 (23.4)				

## DISCUSSION

AKI is a condition in which the kidneys suddenly cannot filter wastes from the blood. It develops rapidly over a few hours or days.^20^ PE-E is one of the 3 leading causes of maternal morbidity and mortality worldwide. During the past 50 years, there has been a significant reduction in the rates of PE-E, maternal mortality, and maternal morbidity in the HICs.^21^ In contrast, the rates of PE-E, and its associated maternal morbidity and mortality remain high in LMICs.^21^

In obstetrics, AKI may result in complications of PE-E, abruptio placenta, uterine haemorrhage, and puerperal sepsis. ^22^ Factors such as parity, age, education, Body Mass Index (BMI), referral status, serum creatinine at admission, protein in the urine, and hypertension have been associated with the development of persistent AKI or worsening AKI. Pathophysiological, PE-E cases are associated with glomerular endothelins which decrease the glomerular filtration rate and renal blood flow.^23^ Furthermore, an increase in vascular resistance predisposes patients to AKI.^23^

In this study, our primary objective was to determine the prevalence of pre-eclampsia/eclamptic-related acute kidney injury and its maternal outcomes among women who were diagnosed with preeclampsia/eclampsia at BMC and Sengerema District Designated Hospital. The study included a total of 258 participants and the cumulative prevalence of AKI was found to be quite high at 54.7%. The level of serum creatinine was found to be the most important independent characteristic on admission, after 72 hours before discharge, or after seven days, depending on which came first. This may be due to patients delaying care until they are admitted to the hospital. Of the patient in our study, 138 (53.5%) were referrals from other facilities, which indicates that the condition may have been present for some time before their arrival. Because we are a tertiary facility, we generally receive PE-E cases in particularly challenging situations. The design of the study puts us in a position to identify PE-E, both of which are associated with increased risk of many problems (multiorgan dysfunction). This magnitude of AKI in this study was much higher than the 10.5% in Ndaboine et al, over a decade ago at BMC among eclamptic-only patients.^15^ The difference was in study population and sample size as compared to the current study which enrolled all pre-eclamptic and eclamptic patients and the sample size was 141 compared to 75 in Ndaboine’s study.^15^ These findings are much higher than those found at Mbarara Regional Referral Hospital by Mariam Hassan et al. 2021 with a prevalence of 41.4%.^24^ This shows that the trend is increasing probably because of the improvement of health referral and reporting systems, lifestyle changes, healthy policy changes, and reporting systems and healthy awareness which cannot be compared with those 10 years back. A study by Ruggajo et al at Dar es Salaam, Tanzania 2022, showed a low prevalence of PRAKI of 8.6% probably because the study involved complications of pregnancy different from our study which completely enrolled PE-E patients only.^25^ However, studies conducted in other developing countries like Cameroon and Morocco, the burden of AKI was 66.7 % and 66.7%.^26,27^ This observation is similar to the study done by Huang C et al., which showed the incidence of AKI in patients with severe preeclampsia as high as 60%.^28^ Jai Prakash et al. also reported that PE/E was responsible for AKI in 43.9 and 35.3 of cases in pregnant women in India.^29^

In this study 4.7% of the participants were HIV positive on ARV and 10 of these had AKI, one-third of them had their AKI completely resolved. This can be explained by the fact that their renal profile was not known before conception, and as it is common knowledge, HIV positivity has been associated with nephropathy which affects the glomerular filtration rate.^30^ This effect, which could be exacerbated by the presence of PE-E, ultimately leads to a more detrimental outcome. It was not possible to determine the severity of the disease, its stage, or the time the participant has been taking ARVs. This was also seen in the study done by Ruggajo et al, at Dar es Salaam Tanzania, which showed PRAKI was associated with HIV positivity.^25^ A study that was done in 2021 at Mbarara Reginal Referral Hospital by Mariam H and colleagues, showed that there was no association between the two, which is in contrast to the findings of this study.^24^

When looking at the AKI stage at admission and its association with maternal outcomes, AKI stage III had a 21-fold risk of ending with poor outcomes. This means that having AKI stage-3 is associated with persistent and worsening outcomes. And for stage II AKI; the risk was high, 2.9-fold. This still shows that having AKI stage-2 is still a risk for persistent ± worsening outcomes. As those who were admitted with high serum creatinine, the majority of them had either persisted with high serum creatinine or worsened to another stage at the end of follow-up on the seventh day, despite of treatment given. This is supported by the studies which show that KDIGO stage-3 was associated with higher maternal mortality.^31^ Three-quarters of the patients with first-stage creatinine levels resolved with conservative management, while approximately one-third of those with second-stage and third-stage creatinine levels required dialysis. This suggests that outcomes were closely related to the severity of the creatinine stage. A limitation of this study was the lack of baseline serum creatinine levels of our participants before pregnancy or at booking, making it difficult to determine if participants had pre-existing renal problems or not which would have been an exclusion criterion. Without further diagnostic tests, such as renal biopsy, it was not possible to identify when the renal insult occurred. Consequently, the severity of the creatinine stage primarily predicted the clinical outcomes.

A previous history of hypertension or PE-E has been associated with renal complications.^3^ AKI and subsequent chronic renal disease can arise as a result of complications of hypertension.^3,32^ This is because chronic hypertension and PE-E causes endothelial injury, which further causes generalised vascular constriction, endothelial injury, and reduced blood flow to vital organs such as kidneys and impair glomerular filtration rate.^32^ For blood pressure at admission, statistical analysis was done on systolic blood pressure or diastolic blood pressure severity which was found statistically not significant on both chi-square and multivariate analysis. The association of systolic blood pressure with AKI in pre-eclamptic/eclamptic patients was seen in a study done by Frances I. et al. 2019.^3^

In the current study, having informal education seems to have an association with poor maternal outcomes. Informal education was found to have 36 odds of poor outcomes. This can be explained by the fact that being educated puts a person in a better position to recognise early danger signs and understand well what is taught in the antenatal clinics, the power to make a decision, and resources to help reach health services earlier.^33^ This means education has an impact on women’s health, as no education was found to be highly associated with worse maternal outcomes. This was observed in some participants during the study period. A study by Nyirenda et al in Zambia in 2019 revealed that higher education attainment empowers women to make choices about their health, including maternal health care.^34^

## Study Limitations

This study was conducted in a referral tertiary centre and one district-designated hospital, and thus the study results cannot be generalised.

Inability to use renal biomarkers such as neutrophil gelatinase-associated lipocalin (NGAL), Interleukin-18 (IL-18), Kidney injury molecule 1(KIM-1), Cystatin C, Urinary microRNA, etc., which are more sensitive for the diagnosis of AKI. This makes it difficult to ascertain the time of onset of renal insult.

Lack of pre-pregnancy or booking antenatal renal function tests made it difficult to know the pre-pregnancy renal status. This makes it difficult to differentiate CKD and AKI among study participants.

## CONCLUSION

The magnitude of acute kidney injury was found to be high among PE-E patients at both BMC and SDDH (54.7%). Advanced AKI stage, HIV-positive status, and informal education level were associated with persistent AKI and worsening AKI.

## RECOMMENDATION

Since PE-E is associated significantly with acute kidney injury (AKI), we strongly advise that the renal profile evaluation should be among the baseline markers for assessment during antenatal care for early recognition and interventions to prevent complications.

Continuous health education at antenatal care (ANC) clinics on danger signs recognition and early seeking habits should be given prompt attention. However, additional research is required to determine the long-term implications of this disease particularly in regions with limited resources.
